# Baseline Psychological Inflexibility Moderates the Outcome Pain Interference in a Randomized Controlled Trial on Internet-based Acceptance and Commitment Therapy for Chronic Pain

**DOI:** 10.3390/jcm8010024

**Published:** 2018-12-25

**Authors:** Thomas Probst, Harald Baumeister, Lance M. McCracken, Jiaxi Lin

**Affiliations:** 1 Department for Psychotherapy and Biopsychosocial Health, Danube University Krems, 3500 Krems, Austria; 2 Department of Clinical Psychology and Psychotherapy, Institute of Psychology, University of Ulm, 89069 Ulm, Germany; harald.baumeister@uni-ulm.de; 3 Psychology Department, Institute of Psychiatry, Psychology & Neuroscience, King’s College London, London WC2R 2LS, UK; lance.mccracken@kcl.ac.uk; 4 INPUT Pain Management Guy’s and St Thomas’ NHS Foundation Trust London, London SE1 7EH, UK; 5 Department of Sports and Sport Science, University of Freiburg, 79098 Freiburg, Germany; jiaxi.lin@sport.uni-freiburg.de

**Keywords:** acceptance and commitment therapy, psychological inflexibility, chronic pain

## Abstract

This study re-investigated data of a randomized controlled trial on Internet-based Acceptance and Commitment Therapy for chronic pain (ACTonPain). Baseline psychological inflexibility was examined as a moderator of the outcome pain interference. In the ACTonPain trial, participants with chronic pain were randomized to one of three conditions: guided Internet-based ACT (*n* = 100), unguided Internet-based ACT (*n* = 101), and waitlist (*n* = 101). Moderation analyses were performed with the SPSS macro PROCESS. Pain interference according to the Multidimensional Pain Inventory (MPI) was the primary outcome in this trial, and the potential moderator psychological inflexibility was measured with the Acceptance and Action Questionnaire (AAQ-II). Psychological inflexibility at baseline moderated the outcome between guided Internet-based ACT and waitlist 9-weeks as well as 6-months after randomization. (both *p* < 0.05). Between unguided Internet-based ACT and waitlist, psychological inflexibility moderated the outcome 6-months after randomization (*p* < 0.05). Internet-based ACT was superior to waitlist for participants with less psychological inflexibility at baseline, but Internet-based ACT became increasingly comparable to waitlist at higher AAQ-II baseline values. Future research should investigate whether the results can be replicated in more individualized and tailored face-to-face settings.

## 1. Introduction 

Chronic pain (CP) is a disorder with a pooled prevalence rate of 31% [1] and it is constantly one of the top causes of years lived with disability [2]. Numerous psychological variables appear to contribute to the process of how pain leads to disability, including self-efficacy, emotional distress, and fear [3]. Psychological therapies have been shown to be effective to improve anxiety, depression, catastrophic thinking, disability, and sometimes also pain [4]. 

A psychotherapy increasingly gaining interest in the treatment of CP is Acceptance and Commitment Therapy (ACT), a contextual form of Cognitive and Behavioral Therapy (CBT). In ACT, psychological flexibility and its opposite psychological inflexibility are central concepts [5]. The six core components of the ACT model of psychological inflexibility are experiential avoidance, cognitive fusion, dominance of the conceptualized past and feared future, attachment to the conceptualized self, lack of values clarity, and inaction, impulsivity, or avoidant persistence. The six core components of the ACT model of psychological flexibility are acceptance, defusion, present moment awareness, self-as-context, values, and committed action. In a meta-analysis, ACT surpassed controls in effects on pain acceptance and psychological flexibility as well as on daily functioning, anxiety, and depression, but not in effects on pain intensity or quality of life [6]. Yet, not all individuals with CP benefit equally as highlighted in a review on predictors of the outcome of contextual CBTs for CP [7]. For example, a recent naturalistic pre-post study on ACT as high intensity, four-week, team-delivered, residential pain management found that higher psychological flexibility predicted better improvements in mental health, whereas lower decentering (reflecting lower cognitive defusion/higher cognitive fusion) predicted better improvements in physical functioning [8]. 

Analyses of predictors of outcomes are important because these identify patient characteristics that influence the outcomes of a specific treatment. Yet, for precision medicine, it is important to know whether one treatment is more or less effective than other treatments or no treatment for participants with specific characteristics. Such interactions between participant and treatment characteristics in the context of a randomized controlled trial (RCT) can be investigated by moderation analyses [9]. The current study is a theoretically-driven investigation of baseline psychological inflexibility as a moderator of the outcome of ACT for CP as suggested by Gilpin and colleagues [7]. Data from the ACTonPain trial [10,11,12] were re-analyzed. In the ACTonPain trial, guided Internet-based ACT for CP, unguided Internet-based ACT for CP, and a waitlist control condition were compared. With regard to the primary outcome pain interference, the two Internet-based ACT conditions did not differ at post-treatment (*p* = 0.99) and follow-up (*p* = 0.99). Guided Internet-based ACT was superior to waitlist at post-treatment (*p* = 0.01) and follow-up (*p* = 0.01). Unguided Internet-based ACT was not superior to waitlist either at discharge (*p* = 0.09) or follow-up (*p* = 0.08). These results were reported by Lin and colleagues [11] and reflect the outcome pain interference for the average participant without taking individual differences into account. The current study took individual differences in psychological inflexibility into account and re-examined the ACTonPain data to explore whether the participants’ baseline psychological inflexibility moderates the primary outcome pain interference between the treatment conditions. 

## 2. Methods

The ACTonPain trial was approved by the ethics committee at the Albert-Ludwigs-University of Freiburg and entered in the German Clinical Trials Registry (DRKS) on 2 October 2014: DRKS00006183. Informed consent was obtained from all participants.

### 2.1. Randomization

In this RCT, N = 302 participants with CP were randomly assigned to one of three conditions. Guided Internet-based ACT (*n* = 100); unguided Internet-based ACT (*n* = 101); and waitlist (*n* = 101; accessed unguided Internet-based ACT after the 6-month follow-up). An independent researcher was responsible for the randomization. Using a web-based randomization program (https://www.sealedenvelope.com/), permuted block randomization with block sizes of 6, 9, and 12 (randomly arranged) was applied (allocation ratio of 1:1:1). 

### 2.2. Participants

Inclusion criteria were chronic pain for at least 6 months, at least 18 years old, at least level two on the von Korff’s pain grading, medically eligible to take part in Internet-based interventions, sufficient knowledge of the German language, sufficient computer and Internet skills, and Internet access [11]. Exclusion criteria were tumor-related pain, ongoing psychological pain treatment or planned treatment in the next three months, and suicidality [11].

The flow diagram and the following percentages (%), means (M), and standard deviations (SD) are published in [11]. On average, the participants were M = 51.7 (SD = 13.1) years old and suffered from pain for M = 114.45 (SD = 121.55) months. Most of the participants were female (84%) and married or with a partner (68%), employed or self-employed (58%). Almost all of the participants had taken part in a treatment for pain in the past (92%). Type of pain was either constant pain with little fluctuation (36%), constant pain with large fluctuations (33%), pain attacks with no pain in between (13%), or pain attacks with pain in between (17%). Pain location was back (34%), head or neck (24%), shoulders (6%), or other (37%). At baseline, the participants of the three conditions did not significantly differ from each other (see [11]). At the 6-month follow-up, between 26% and 46% of the participants had incomplete data [11].

### 2.3. Treatment

The guided and unguided Internet-based ACT conditions comprised the same seven treatment modules (one module per week, approximately 60 min per module). These modules were developed to reduce psychological inflexibility and to increase psychological flexibility. The modules and the corresponding ACT processes of psychological flexibility were as follows. (1) Welcome—present moment awareness. (2) Control and acceptance—present moment awareness, acceptance. (3) Thoughts and emotions—present moment awareness, defusion, values. (4) You and your self—present moment awareness, self-as-context, values. (5) What I value in life—present moment awareness, values. (6) Commitment—present moment awareness, acceptance, committed action. (7) Looking ahead—present moment awareness, values. 

The modules were newer versions of the modules developed by Buhrmann and colleagues [13]. All of them consisted of information, assignments, relevant metaphors, and mindfulness exercises. Intervention materials were provided as integral parts of each module. A read-aloud function was integrated so that the participants could use the audio-narration of each module. The content was presented in short paragraphs, tables, illustrations, pictures, and videos. Furthermore, three characteristic examples of individuals with chronic pain were presented as vignettes, which accompanied the participants in all modules to enable observational learning.

Both Internet-based ACT conditions were identical except that trained psychologists (supervised by H.B.) provided personalized and standardized feedback, reinforcement, and reminders only in guided Internet-based ACT. The psychologists sent an e-mail within two working days after completion of each module and the total time for guidance amounted to an average of 105 min per participant. Participants discontinuing the intervention were *n* = 40 in the guided condition and *n* = 61 in unguided condition [11].

### 2.4. Measures

Pain interference scale of the Multidimensional Pain Inventory (MPI [14]; German version [15]). The primary outcome in the ACTonPain trial was pain interference assessed with the pain interference scale of the German version of the MPI. This scale has 10 items and was administered online at baseline (t0), 9-weeks (t1), and 6-months (t2) after randomization. Higher values indicate more severe pain interference. The MPI items were rated from 0 to 6. Internal consistency amounts to *α* = 0.94 and the test–retest correlation is *r* = 0.78.

Acceptance and Action Questionnaire (AAQ-II [16]; German version [17]). Psychological inflexibility was measured online with the German version of the AAQ-II. Higher scores stand for higher psychological inflexibility. The AAQ-II items were scored from 0 to 6. The internal consistency ranges from *α* = 0.84 to *α* = 0.97. Test-retest reliability is between *r* = 0.74 and *r* = 0.85.

### 2.5. Statistics

The moderation analyses were performed with PROCESS (v3.1 [18]). 

The first two simple moderation models investigated whether baseline psychological inflexibility moderates the outcome pain interference between guided Internet-based ACT and waitlist. Pain interference at t1 (first model)/at t2 (second model) was the outcome variable (*Y*-variable), pain interference at t0 functioned as a covariate, psychological inflexibility at t0 was the potential moderator (*W*-variable), and the independent variable (*X*-variable) was guided Internet-based ACT vs. waitlist. 

Two further simple moderation models explored baseline psychological inflexibility as moderator of the outcome pain interference between unguided Internet-based ACT and waitlist. Pain interference at t1 (first model)/at t2 (second model) was again the outcome variable (*Y*-variable), pain interference at t0 was added as a covariate, psychological inflexibility at t0 functioned as the potential moderator (*W*-variable), and the independent variable (*X*-variable) was the unguided Internet-based ACT vs. waitlist.

In the last two simple moderation models, the focus was on the moderating effects of baseline psychological inflexibility on the outcome pain interference between guided Internet-based ACT and unguided Internet-based ACT. Again, pain interference at t1 (first model)/at t2 (second model) functioned as the outcome variable (*Y*-variable), pain interference at t0 was added as a covariate, psychological inflexibility at t0 as the potential moderator (*W*-variable), and guided Internet-based ACT vs. unguided Internet-based ACT as the independent variable (*X*-variable).

A statistically significant interaction between the *X*-variable and the *W*-variable indicate a moderation effect. Such an effect was further analyzed by the Johnson–Neyman Technique. This technique reveals the threshold(s) of the moderator where the association between the independent variable and the outcome transition(s) between statistical significance and non-significance. As in other publications on the results of the ACTonPain trial [11,12], missing data were handled with the expectation maximization algorithm (EM). 

Moreover, we calculated correlations (Pearson correlation coefficients) between baseline psychological inflexibility and the number of completed treatment modules in Internet-based ACT. A significant correlation would indicate that baseline psychological inflexibility might influence the outcome through treatment adherence.

The significance value was set to *p* <0.05.

## 3. Results

### 3.1. Guided Internet-based ACT vs. Waitlist

**Figure 1:**
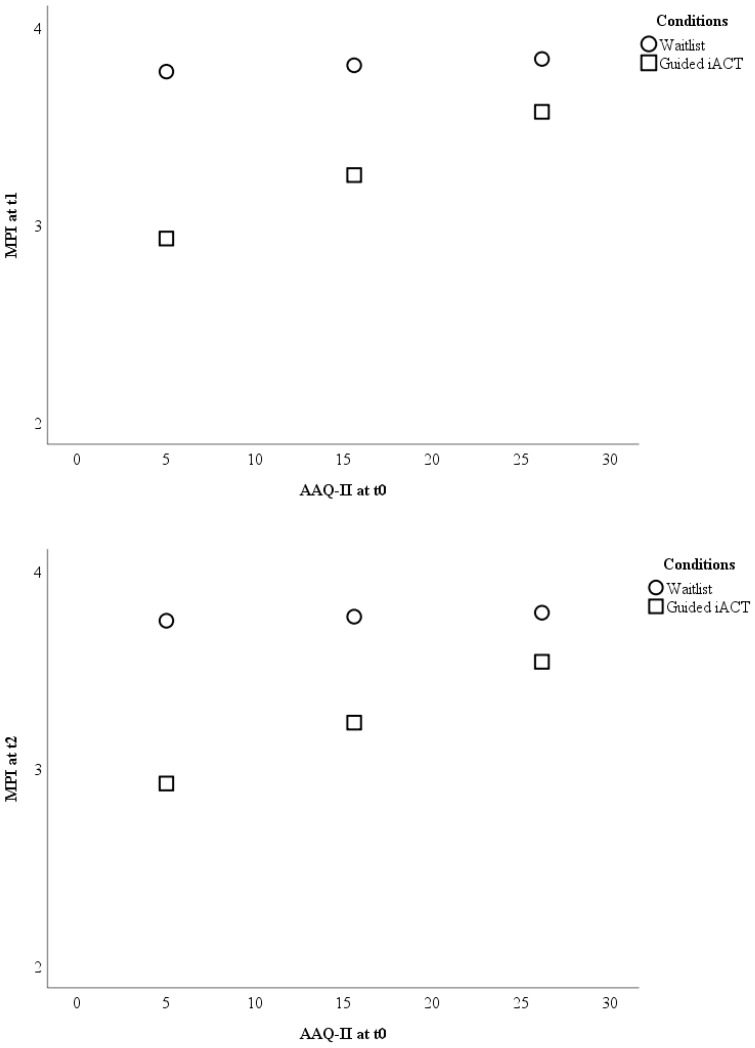
Visualizations of the results testing baseline psychological inflexibility as a moderator of the treatment outcome pain interference between waitlist and guided Internet-based ACT. The outcome (*Y*-axis) is displayed for three different levels (sample mean ± 1 standard deviation) of psychological inflexibility (*X*-axis). Note: AAQ-II = Acceptance and Action Questionnaire; iACT = Internet-based Acceptance and Commitment Therapy; MPI = Pain interference scale of the Multidimensional Pain Inventory; t0 = baseline; t1 = 9-weeks after randomization; t2 = 6-months after randomization.

Baseline psychological inflexibility moderated the outcome between guided Internet-based ACT and waitlist at t1 (F (1; 196) = 7.74; *p* = 0.006) as well as t2 (F (1; 196) = 5.93; *p* = 0.016). Guided Internet-based ACT was superior to waitlist when participants had less psychological inflexibility at baseline (see Figure 1). The outcome at t1 was better for guided Internet-based ACT than for waitlist in participants with AAQ-II baseline scores <25.70. At t2, online ACT surpassed waitlist in participants with AAQ-II baseline scores <24.32. However, the outcome of Internet-based ACT and waitlist became increasingly comparable at higher AAQ-II baseline values (see Figure 1). At t1, the outcome was comparable in participants with AAQ-II scores ≥25.70. The outcome at t2 was comparable in participants with AAQ-II scores ≥24.32. 

### 3.2. Unguided Internet-based ACT vs. Waitlist

**Figure 2:**
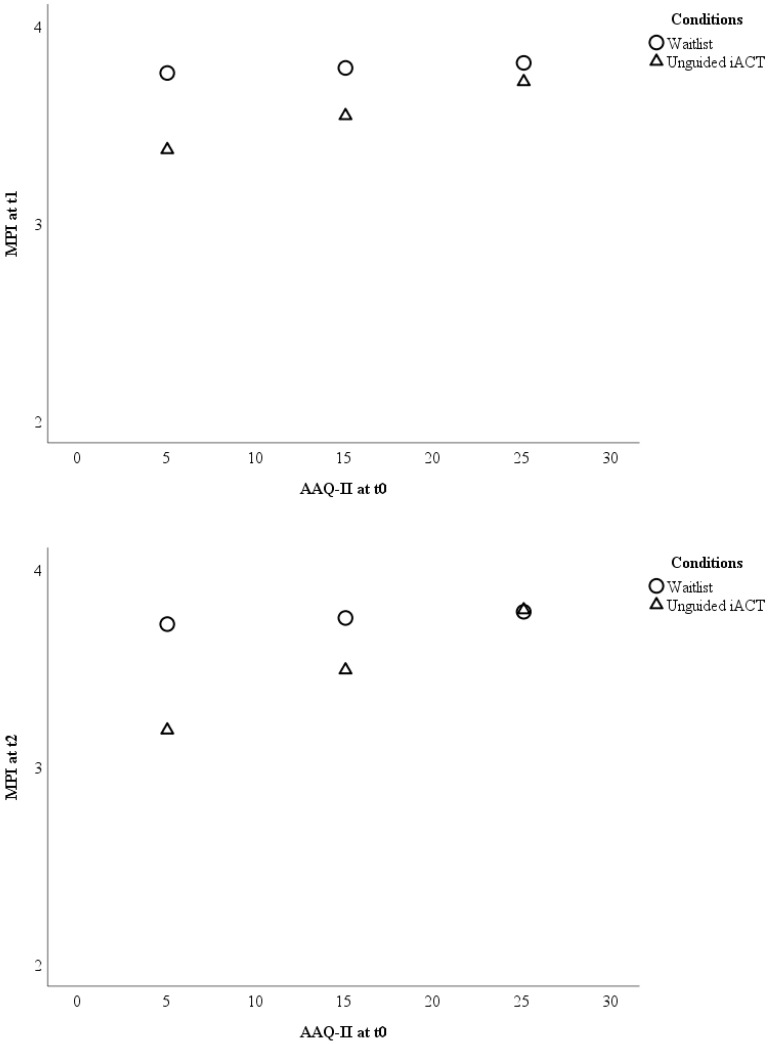
Visualizations of the results testing baseline psychological inflexibility as a moderator of the treatment outcome pain interference between waitlist and unguided Internet-based ACT. The outcome (*Y*-axis) is displayed for three different levels (sample mean ± 1 standard deviation) of psychological inflexibility (*X*-axis). Note: AAQ-II = Acceptance and Action Questionnaire; iACT = Internet-based Acceptance and Commitment Therapy; MPI = Pain interference scale of the Multidimensional Pain Inventory; t0 = baseline; t1 = 9-weeks after randomization; t2 = 6-months after randomization.

Baseline psychological inflexibility moderated the outcome between unguided Internet-based ACT and waitlist at t2 (F (1; 197) = 4.93; *p* = 0.028) but not at t1 (F (1; 197) = 2.09; *p* = 0.150). At t2, participants with AAQ-II scores <15.93 benefited more from unguided Internet-based ACT than from waitlist, whereas the outcome was comparable in participants with AAQ-II scores ≥15.93 (see Figure 2).

### 3.3. Guided vs. Unguided Internet-based ACT

**Figure 3:**
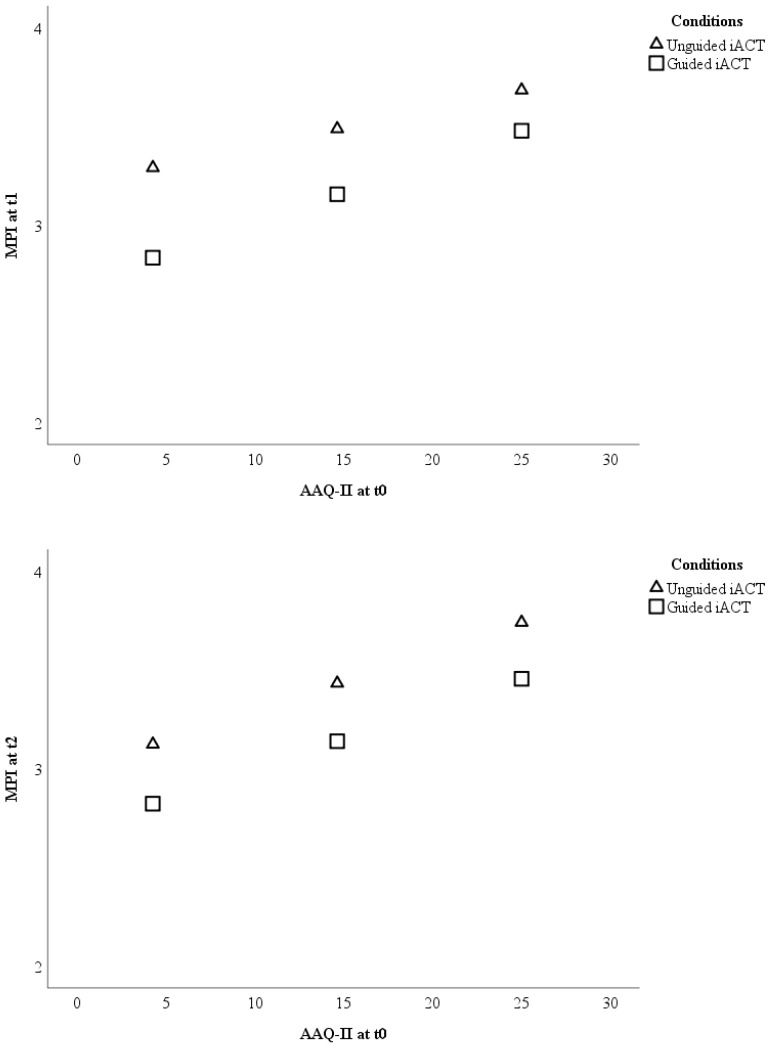
Visualizations of the results testing baseline psychological inflexibility as a moderator of the treatment outcome pain interference between unguided and guided Internet-based ACT. The outcome (*Y*-axis) is displayed for three different levels (sample mean ± 1 standard deviation) of psychological inflexibility (*X*-axis). Note: AAQ-II = Acceptance and Action Questionnaire; iACT = Internet-based Acceptance and Commitment Therapy; MPI = Pain interference scale of the Multidimensional Pain Inventory; t0 = baseline; t1 = 9-weeks after randomization; t2 = 6-months after randomization.

Psychological inflexibility at baseline did not moderate the outcome between guided and unguided Internet-based ACT (see Figure 3) either at t1 (F (1; 196) = 1.30; *p* = 0.256) or at t2 (F (1; 196) = 0.004; *p* = 0.951).

### 3.4. Relationship between Psychological Inflexibility and Completed Treatment Modules

One might hypothesize that the outcome of Internet-based ACT became comparable to waitlist for participants with higher baseline psychological inflexibility because these participants completed fewer treatment modules than participants with lower baseline psychological inflexibility. The Pearson correlation coefficients between the baseline AAQ-II scores and the number of completed treatment modules did, however, not reach statistical significance either in guided Internet-based ACT (*r* = −0.03; *p* = 0.735) or in unguided Internet-based ACT (*r* = 0.02; *p* = 0.881).

## 4. Discussion

As shown in a previous publication, guided Internet-based ACT for CP surpassed waitlist but unguided Internet-based ACT for CP did not for the average participant in the ACTonPain trial [11]. Yet, differential results emerged when including baseline psychological inflexibility as a moderator. Guided Internet-based ACT surpassed waitlist at post-treatment and follow-up in participants with less baseline psychological inflexibility, but not in participants with higher baseline psychological inflexibility. Comparably, unguided Internet-based ACT was superior to waitlist at follow-up in participants with less baseline psychological inflexibility, but not in participants with higher baseline psychological inflexibility. These results show how important it is to consider the moderating role of specific client characteristics, such as baseline psychological inflexibility, when exploring the outcome of clinical trials. As the number of completed treatment modules was not associated with psychological inflexibility, it can be ruled out that psychologically inflexible participants showed a comparable outcome in waitlist and Internet-based ACT just because they completed fewer treatment modules than psychologically flexible participants did.

In summary, baseline psychological inflexibility moderated the outcome, yet, not in favor of ACT. This result is in line with studies comparing ACT and CBT in patients with anxiety disorders, which also found that baseline psychological inflexibility moderates the outcome not in favor of ACT [19,20]. 

If our results can be replicated, practical implications would be as follows: Internet-based ACT should be recommended over no treatment for individuals with CP who are psychologically flexible at baseline. For individuals with CP and higher psychological inflexibility at baseline, however, Internet-based ACT should not be preferred over no treatment. Applying additional treatment methods to increase psychological flexibility before Internet-based ACT might help to deliver its full potential for high psychologically inflexible individuals. For example, adding more individualized and tailored face-to-face interventions might be of particular benefit for the individuals who are psychologically inflexible. However, a recent study found that also in face-to-face ACT—provided as high intensity, team-delivered, residential pain management—psychologically inflexible individuals did not benefit as much as psychologically flexible patients regarding mental health [8]. Further studies are necessary to address whether face-to-face psychotherapies are more suited than Internet-based treatments for psychologically inflexible patients. Although Internet-based and face-to-face psychotherapies seem to show comparable effects [21] also for CP [22], patient variables can moderate the outcomes between these delivery formats (e.g., [23]). As we used a global measure of psychological inflexibility, future research might also investigate which specific aspect of psychological inflexibility moderates treatment outcomes in ACT for CP (experiential avoidance, cognitive fusion, dominance of the conceptualized past and feared future, attachment to the conceptualized self, lack of values clarity, and inaction, impulsivity, or avoidant persistence). Gilpin and colleagues [8] reported that different aspects of baseline psychological inflexibility exert different effects on outcomes in ACT for CP. While higher baseline psychological flexibility predicted a better outcome in mental health, lower decentering (reflecting lower cognitive defusion/higher cognitive fusion) predicted better improvements in physical functioning. Taken together, the results of the ACTonPain trial indicate that an Internet-based ACT for CP is efficacious [11]. Increases in psychological flexibility are associated with an improved outcome [12], however, the study at hand shows that it requires a certain amount of psychological flexibility already at baseline to benefit from Internet-based ACT for CP. Therefore, screening for psychological inflexibility before starting Internet-based ACT for CP appears to be necessary so that only the individuals with CP that are likely to benefit are assigned to Internet-based ACT.

## References

[1] Steingrímsdóttir Ó.A., Landmark T., Macfarlane G.J., Nielsen C.S. (2017). Defining chronic pain in epidemiological studies: A systematic review and meta-analysis. Pain.

[2] GBD 2016 Disease and Injury Incidence and Prevalence Collaborators (2017). Global, Regional, and National Incidence, Prevalence, and Years Lived with Disability for 328 Diseases and Injuries for 195 Countries, 1990–2016: A Systematic Analysis for the Global Burden of Disease Study 2016. Lancet.

[3] Lee H., Hübscher M., Moseley G.L., Kamper S.J., Traeger A.C., Mansell G., McAuley J.H. (2015). How does pain lead to disability? A systematic review and meta-analysis of mediation studies in people with back and neck pain. Pain.

[4] Williams A.C., Eccleston C., Morley S. (2012). Psychological therapies for the management of chronic pain (excluding headache) in adults. Cochrane Database Syst. Rev..

[5] Hayes S.C., Luoma J.B., Bond F.W., Masuda A., Lillis J. (2006). Acceptance and commitment therapy: Model, processes and outcomes. Behav. Res. Ther..

[6] Hughes L.S., Clark J., Colclough J.A., Dale E., McMillan D. (2017). Acceptance and Commitment Therapy (ACT) for chronic pain: A systematic review and meta-analyses. Clin. J. Pain.

[7] Gilpin H.R., Keyes A., Stahl D.R., Greig R., McCracken L.M. (2017). Predictors of treatment outcome in contextual cognitive and behavioral therapies for chronic pain: A systematic review. J. Pain.

[8] Gilpin H.R., Stahl D.R., McCracken L.M. (2018). A theoretically guided approach to identifying predictors of treatment outcome in contextual CBT for chronic pain. Eur. J. Pain.

[9] Van Hoorn R., Tummers M., Booth A., Gerhardus A., Rehfuess E., Hind D., Bossuyt P.M., Welch V., Debray T.P., Underwood M. (2017). The development of CHAMP: A checklist for the appraisal of moderators and predictors. BMC Med. Res. Methodol..

[10] Lin J., Lüking M., Ebert D.D., Buhrman M., Andersson G., Baumeister H. (2015). Effectiveness and cost-effectiveness of a guided and unguided internet-based Acceptance and Commitment Therapy for chronic pain: Study protocol for a three-armed randomised controlled trial. Internet Interv..

[11] Lin J., Paganini S., Sander L., Lüking M., Ebert D.D., Buhrman M., Andersson G., Baumeister H. (2017). An Internet-based intervention for chronic pain—A three-arm randomized controlled study of the effectiveness of guided and unguided acceptance and commitment therapy. Deutsch. Ärztebl. Int..

[12] Lin J., Klatt L.-I., McCracken L.M., Baumeister H. (2018). Psychological flexibility mediates the effect of an online-based acceptance and commitment therapy for chronic pain. Pain.

[13] Buhrman M., Skoglund A., Husell J., Bergström K., Gordh T., Hursti T., Bendelin N., Furmark T., Andersson G. (2013). Guided internet-delivered acceptance and commitment therapy for chronic pain patients: A randomized controlled trial. Behav. Res. Ther..

[14] Kerns R.D., Turk D.C., Rudy T.E. (1985). The West Haven-Yale Multidimensional Pain Inventory (WHYMPI). Pain.

[15] Flor H., Rudy T.E., Birbaumer N., Streit B., Schugens M.M. (1990). Zur Anwendbarkeit des West Haven-Yale multidimensional pain inventory im Deutschen Sprachraum. Der Schmerz.

[16] Bond F.W., Hayes S.C., Baer R.A., Carpenter K.M., Guenole N., Orcutt H.K., Waltz T., Zettle R.D. (2011). Preliminary psychometric properties of the Acceptance and Action Questionnaire-II: A revised measure of psychological inflexibility and experiential avoidance. Behav. Ther..

[17] Hoyer J., Gloster A.T. (2013). Psychologische Flexibilität messen: Der Fragebogen zu Akzeptanz und Handeln II. Verhaltenstherapie.

[18] Hayes A.F. (2018). Introduction to Mediation, Moderation, and Conditional Process Analysis.

[19] Wolitzky-Taylor K.B., Arch J.J., Rosenfield D., Craske M.G. (2012). Moderators and non-specific predictors of treatment outcome for anxiety disorders: A comparison of cognitive behavioral therapy to acceptance and commitment therapy. J. Consult. Clin. Psychol..

[20] Craske M.G., Niles A.N., Burklund L.J., Wolitzky-Taylor K.B., Vilardaga J.C.P., Arch J.J., Saxbe D.E., Lieberman M.D. (2014). Randomized controlled trial of cognitive behavioral therapy and acceptance and commitment therapy for social phobia: Outcomes and moderators. J. Consult. Clin. Psychol..

[21] Carlbring P., Andersson G., Cuijpers P., Riper H., Hedman-Lagerlöf E. (2018). Internet-based vs. face-to-face cognitive behavior therapy for psychiatric and somatic disorders: an updated systematic review and meta-analysis. Cogn. Behav. Ther..

[22] Buhrman M., Gordh T., Andersson G. (2016). Internet interventions for chronic pain including headache: A systematic review. Internet Interv..

[23] Kleinstäuber M., Weise C., Andersson G., Probst T. (2018). Personality traits predict and moderate the outcome of Internet-based cognitive behavioural therapy for chronic tinnitus. Int. J. Audiol..

